# Supplementary material for: Spatial and temporal dynamics of SAR11 marine bacteria across a nearshore to offshore transect in the tropical Pacific Ocean
Source: PeerJ. 2021 Nov 4;9:e12274. doi: 10.7717/peerj.12274 (PMC8572523; doi:10.7717/peerj.12274)
Supplement: Supplemental Information 2 [file peerj-09-12274-s002.pdf]

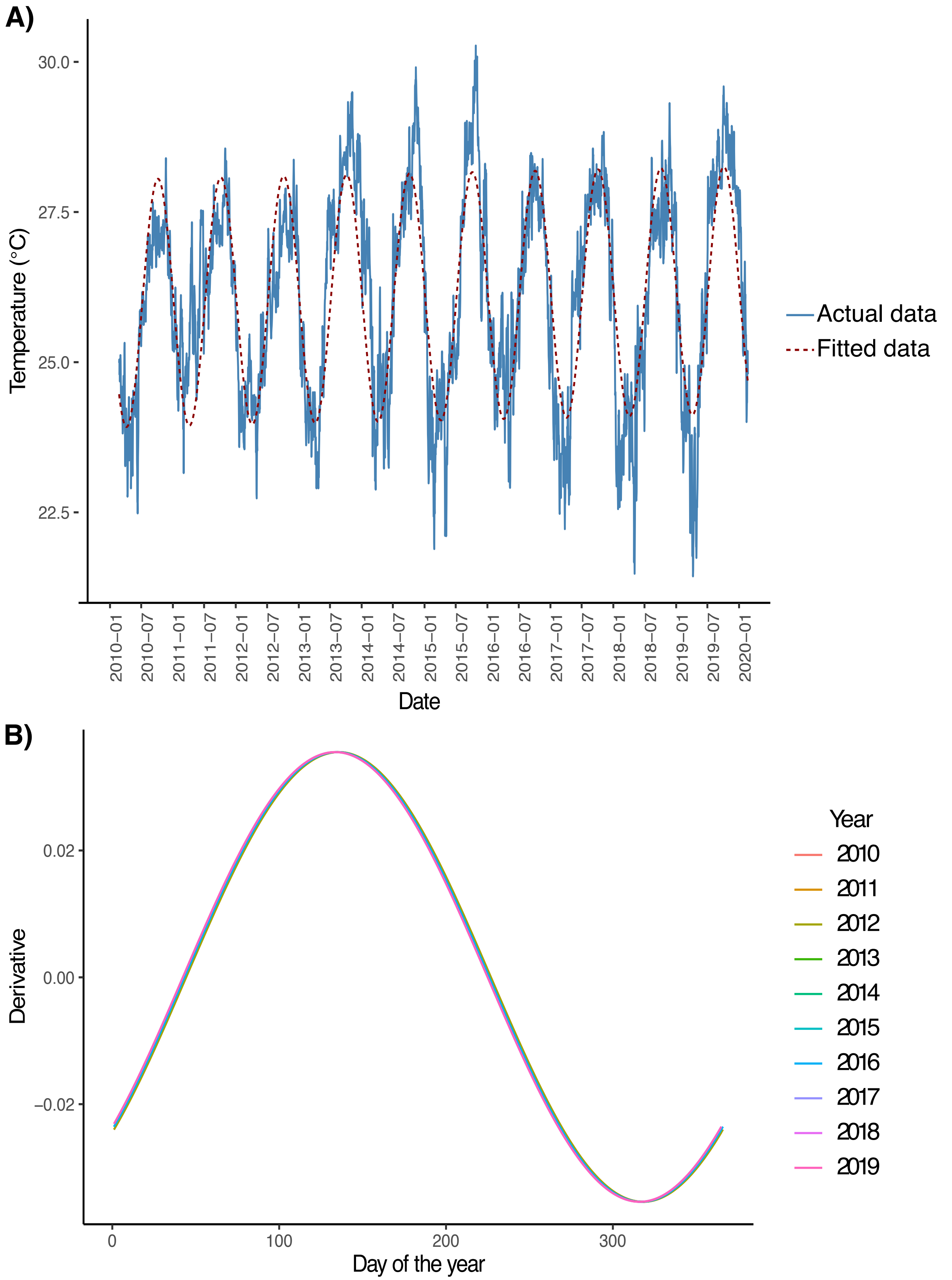

Figure S1. Delineating seasons in KByT. (A) Harmonic mean function fit to sea surface water temperature from Kāne‘ohe Bay, Hawai‘i. **(B)** Annual time-derivate for all 10 years of data.

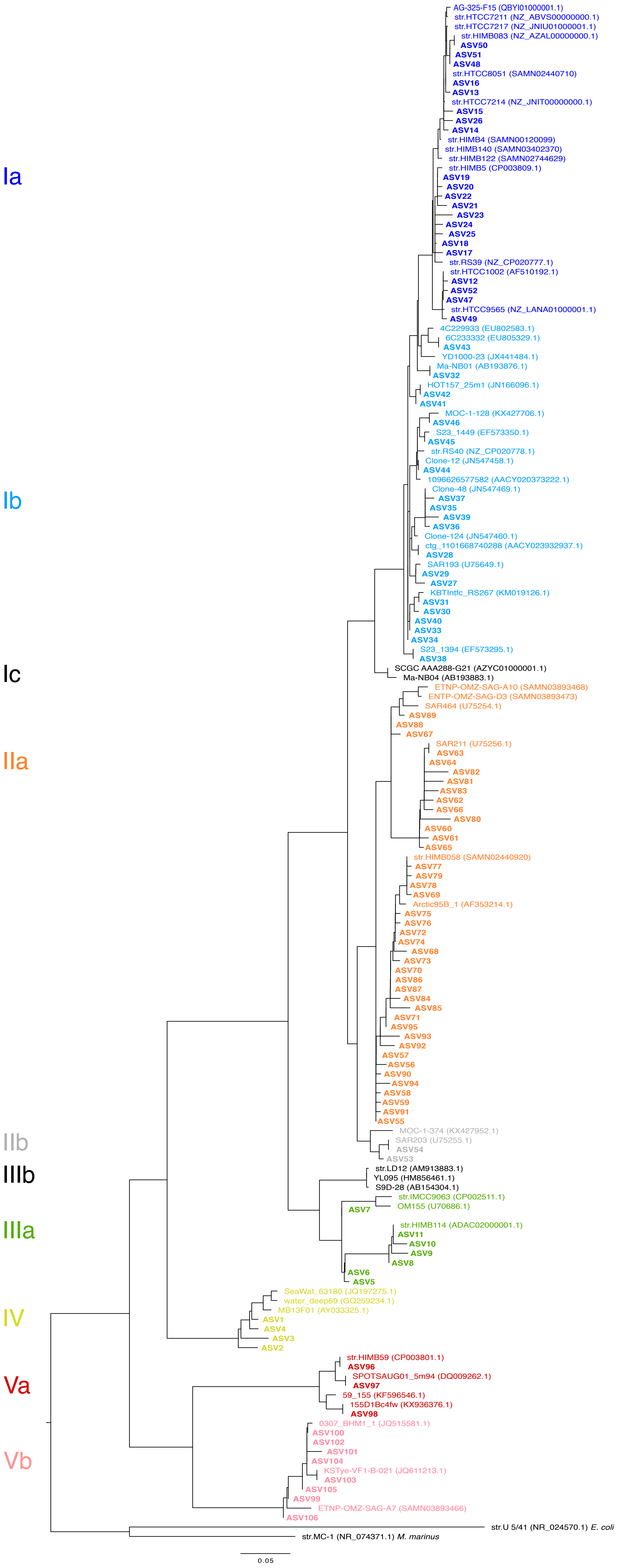

Figure S2. Phylogenetic analysis of SAR11 16S rRNA gene ASVs recovered through KByT. Cultivated isolates are indicated by “str.” (strain). Genbank accession numbers for reference sequences are included in parentheses.

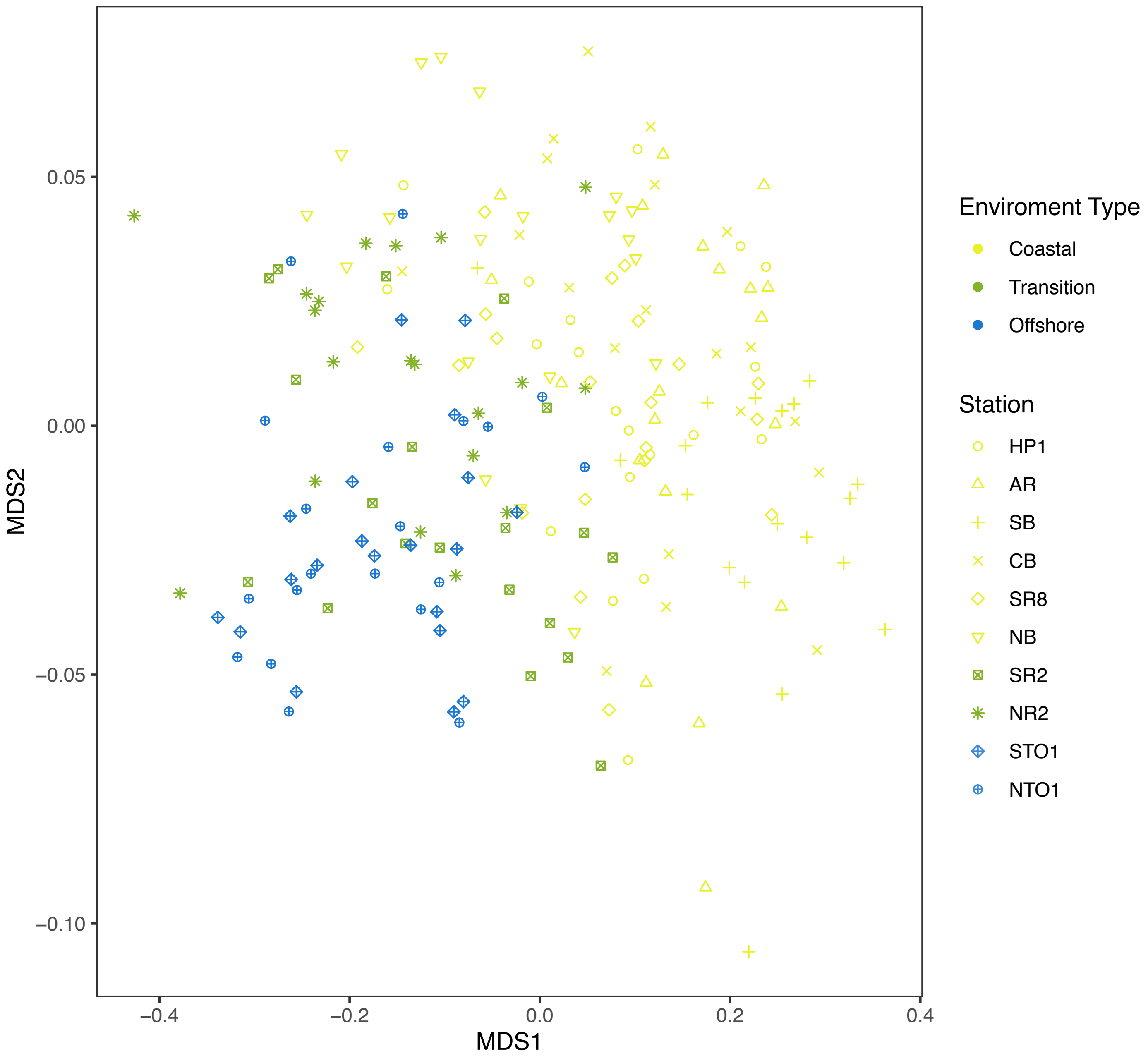

Figure S3. Delineating environment types in KByT. nMDS (k=2) of a Bray-Curtis distance matrix calculated from temperature, salinity, pH, cellular abundances of heterotrophic bacteria, cyanobacteria, and eukaryotic picophytoplankton, and chlorophyll-a concentrations for the entire KByT dataset (n=200 samples). nMDS stress was 0.03.

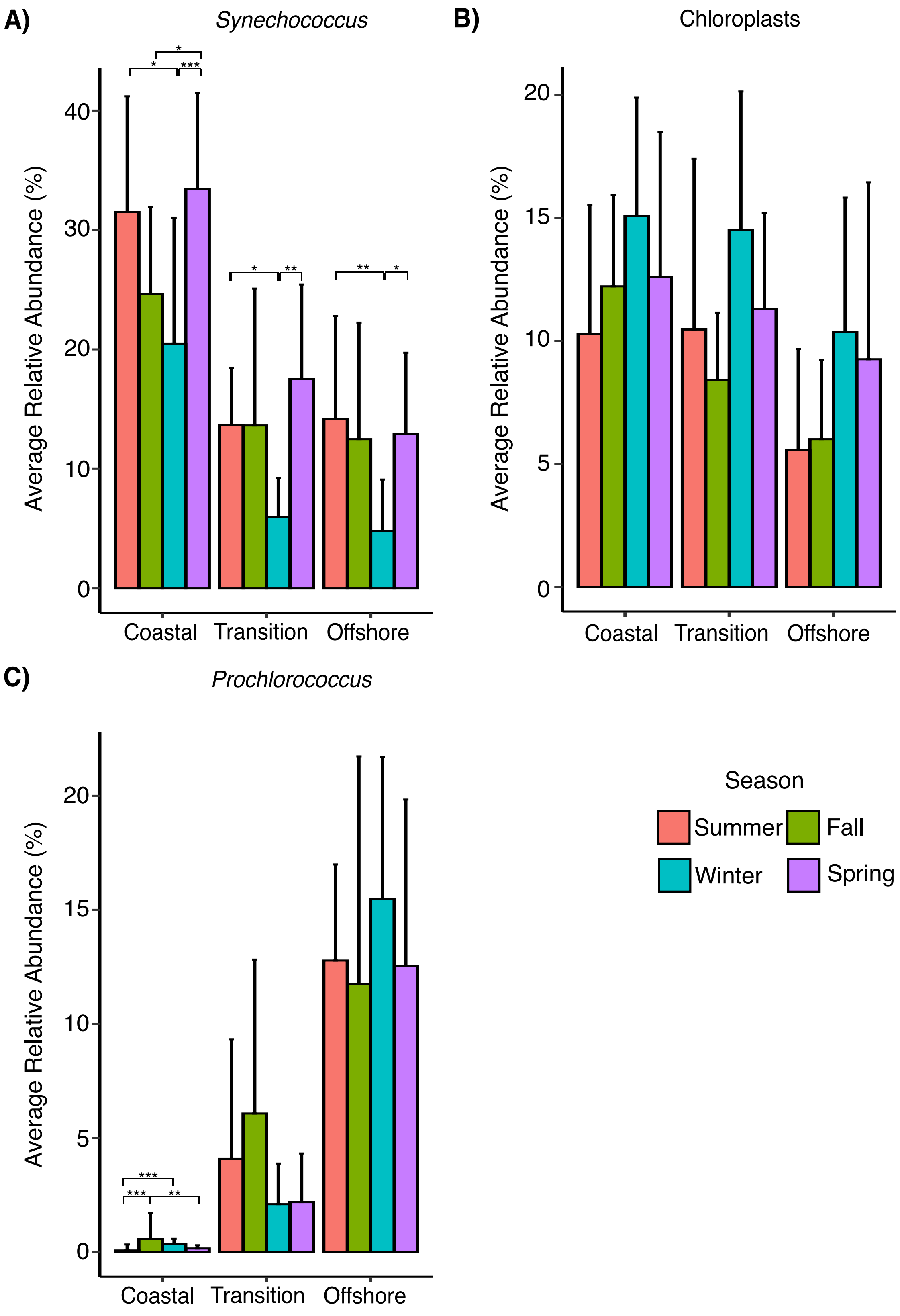

Figure S4. Seasonal and environmental distributions of dominant photoautotrophs in KByT. Average abundance and standard deviation of (**A**) *Synechococcus*, (**B**) chloroplast, and (**C**) *Prochlorococcus* sequences relative to the total community in each environment and season. Seasonal comparisons with significant seasonal differences as defined by DESeq2 normalization are noted: * if $p<0.05$, ** if $p\leq0.01$, and *** if $p\leq 0.001$, while non-significant comparisons are not noted.

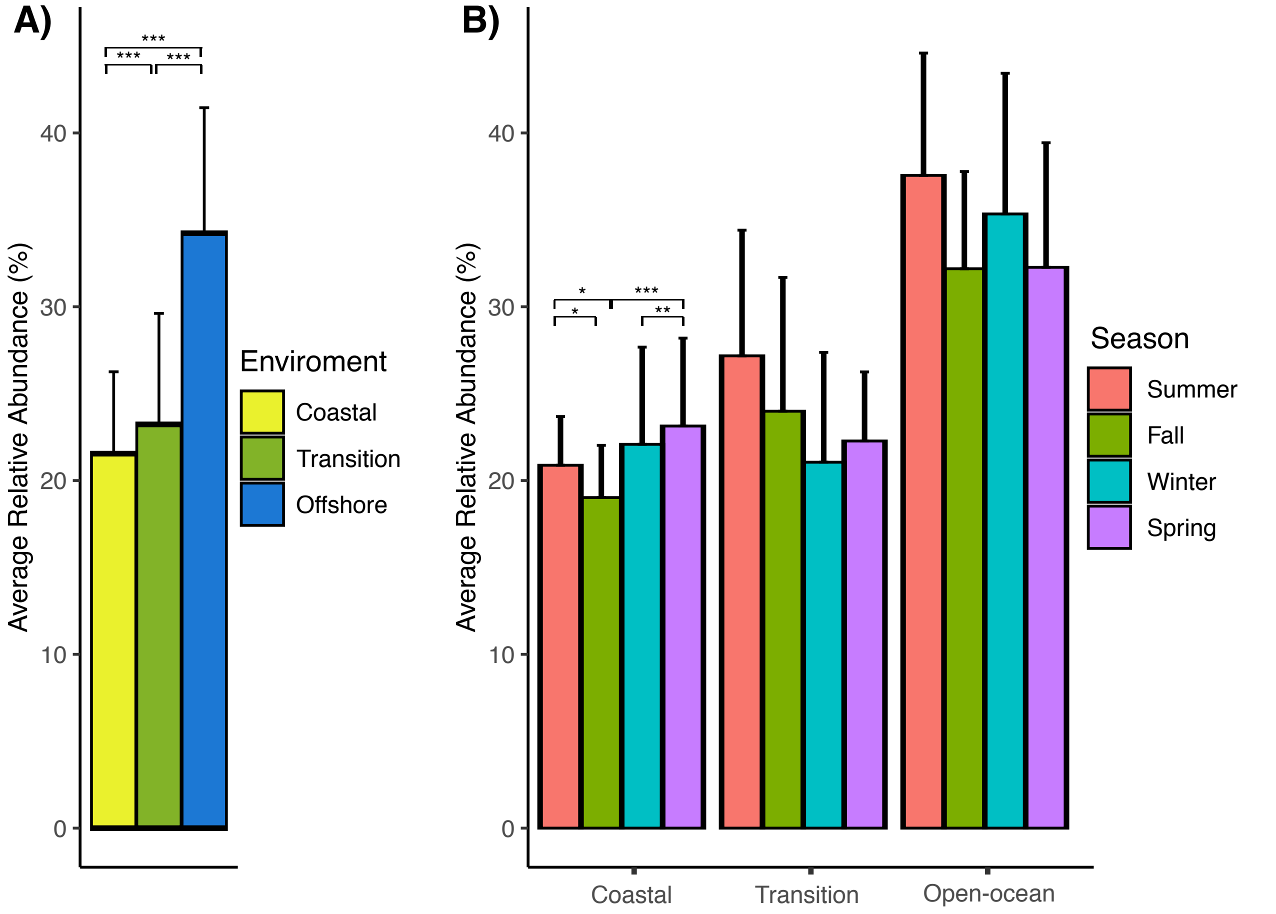

Figure S5. Distribution of total SAR11 relative abundance in KByT. (A) Average abundance and standard deviation of SAR11 relative to the total community in each environment. **(B)** Average abundance and standard deviation of SAR11 relative to the total community in each season. Comparisons with significant seasonal or environmental differences as defined by DESeq2 normalization are noted: * if $p<0.05$, ** if $p\leq0.01$, and *** if $p\leq 0.001$, while non-significant comparisons are not noted.

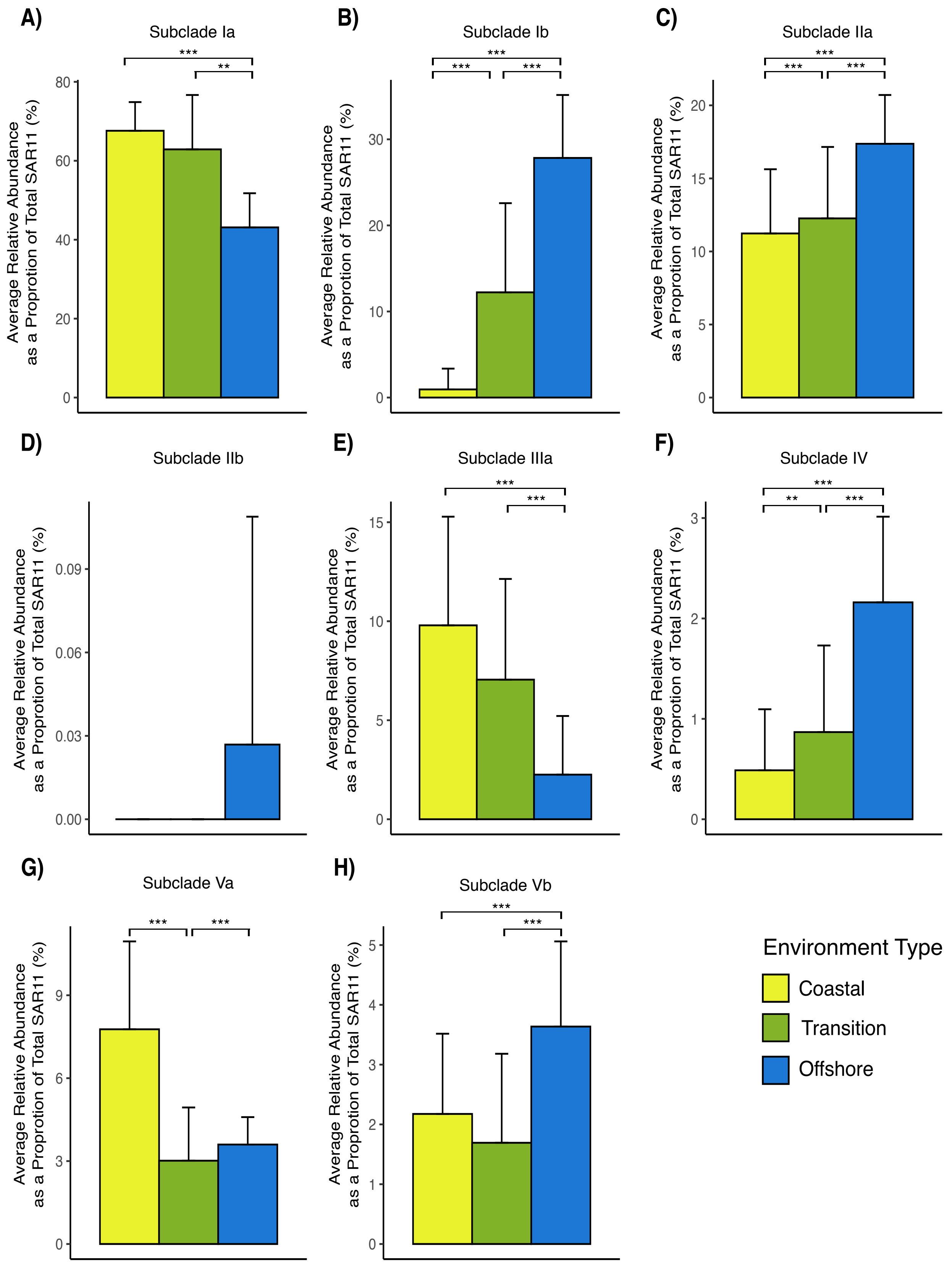

Figure S6. Environmental distributions of SAR11 subclades in KByT. Average abundance and standard deviation of SAR11 subclades relative to total SAR11 abundance in each environment. Comparisons with significant environmental differences as defined by DESeq2 normalization are noted: * if $p<0.05$, ** if $p\leq0.01$, and *** if $p\leq 0.001$, while non-significant comparisons are not noted.

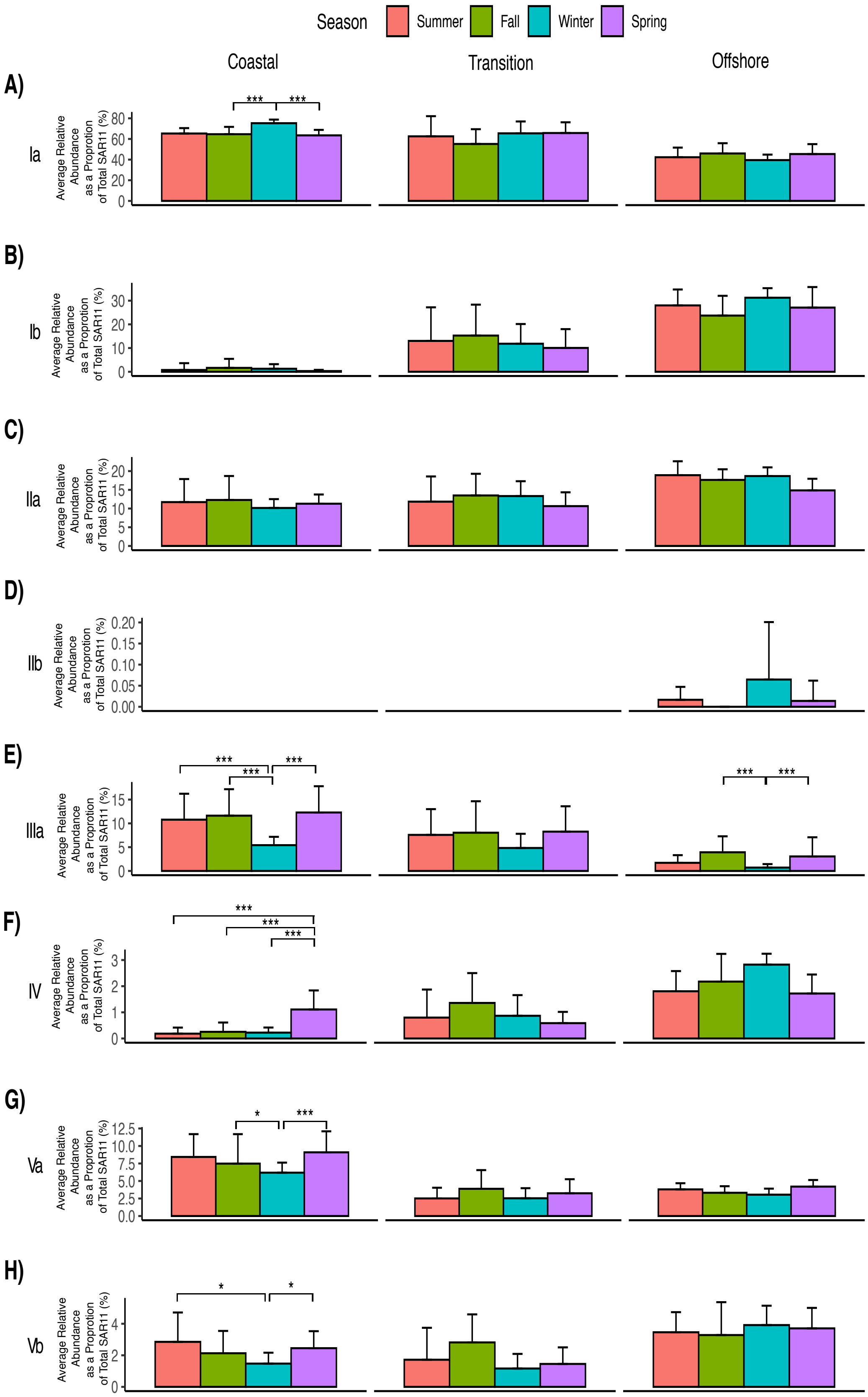

Figure S7. Seasonal distributions of SAR11 subclades in KByT. Average abundance and standard deviation of SAR11 subclades relative to total SAR11 abundance in each environment (coastal, transition, offshore) per season. Seasonal comparisons with significant seasonal differences as defined by DESeq2 normalization are noted: * if p<0.05, ** if p≤0.01, and *** if p≤ 0.001, while non-significant comparisons are not noted.

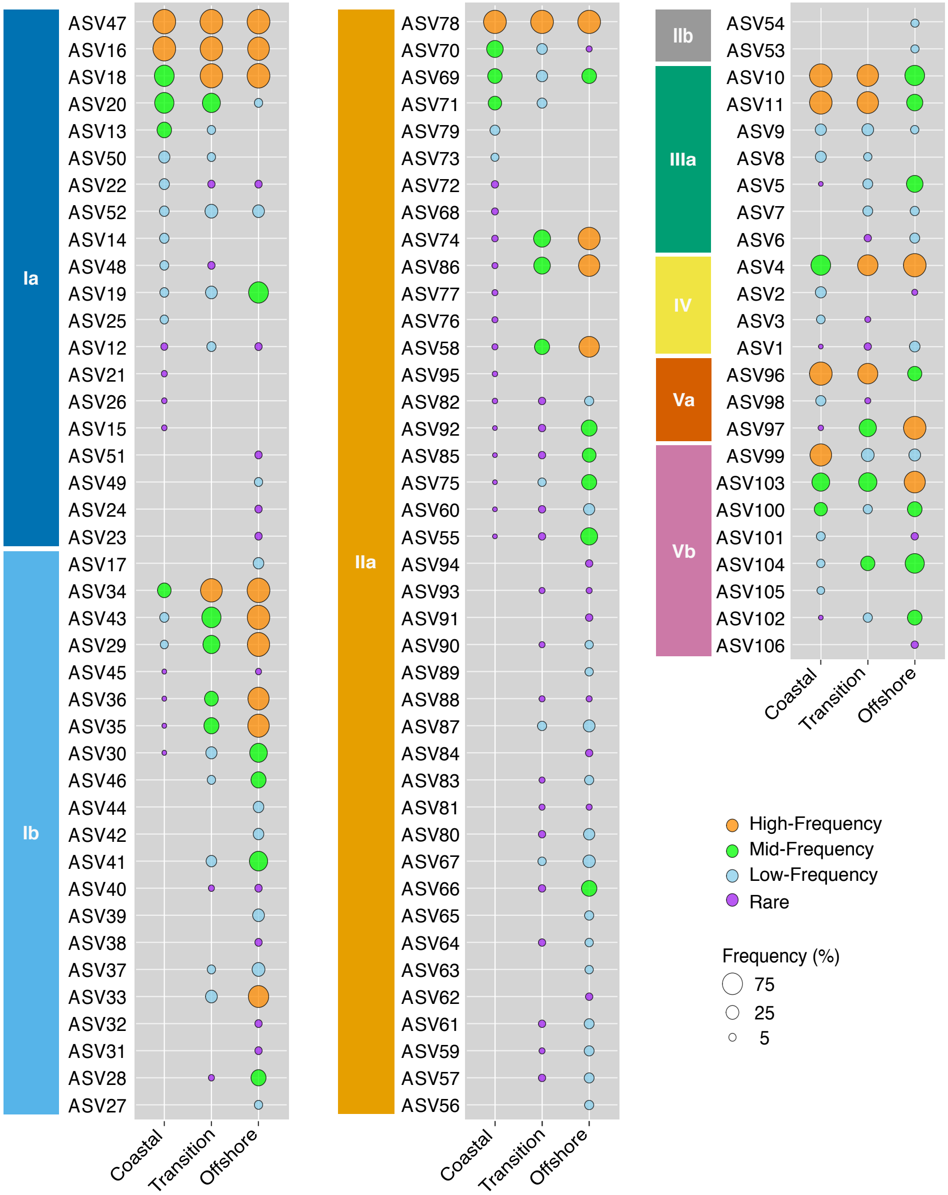

Figure S8. Frequency of detection of SAR11 ASVs across subclades and environments. Sample size per environment of KByT included coastal (n=120), transition (n=40), and offshore (n=40).

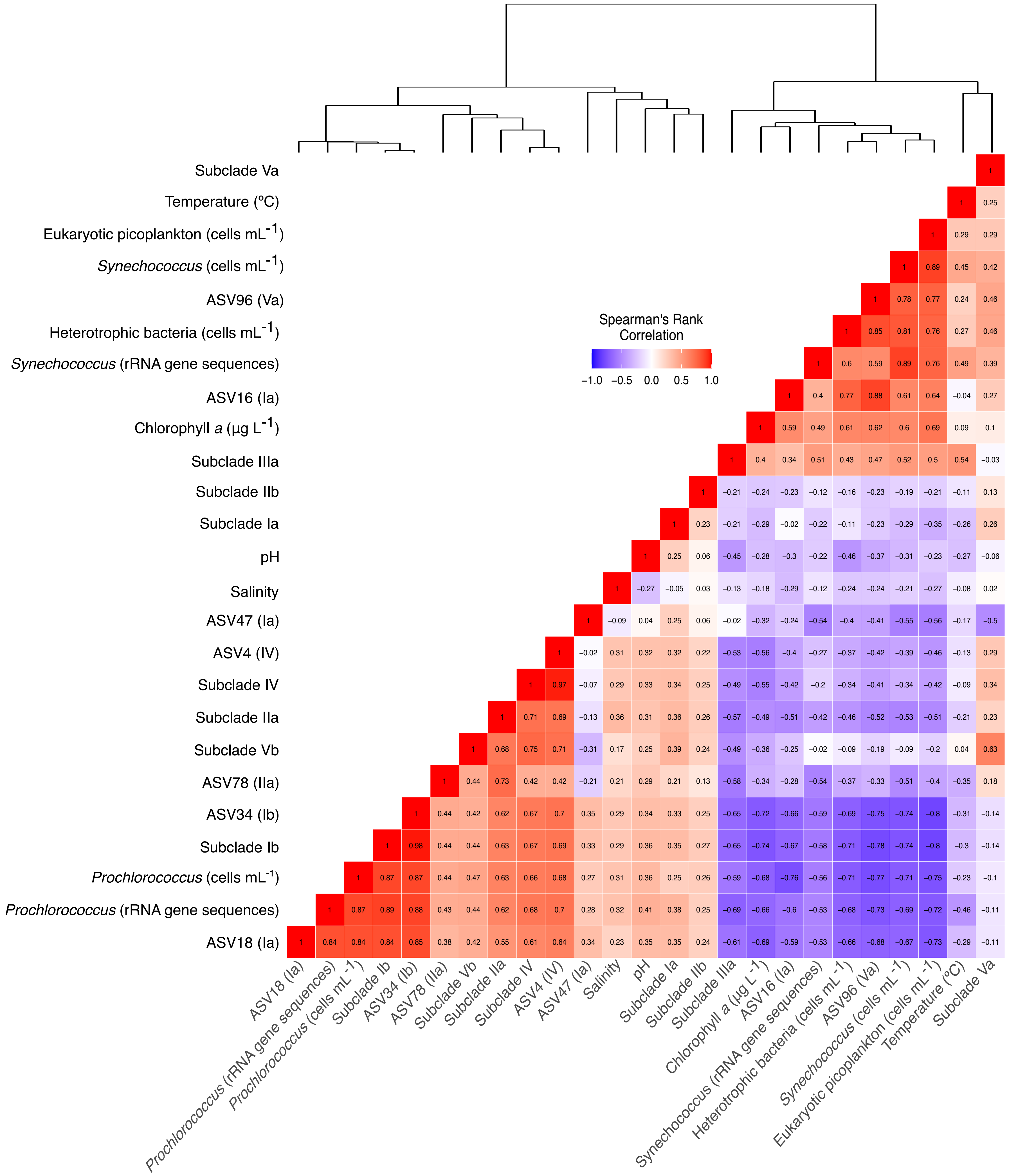

Figure S9. Correlation analysis of SAR11 subclades, dominant SAR11 ASVs, picocyanobacteria, and environmental parameters. Spearman's Rank correlation coefficients between variance stabilized transformed DESeq2 normalized abundances of SAR11 subclades, dominant SAR11 ASVs, and picocyanobacterial and environmental parameters are shown. The dendrogram was built from a matrix of the correlation coefficients, clustering these correlated variables into two main groups- those associated to offshore conditions and those associated to coastal conditions.
